# Diverse associations between pancreatic intra-, inter-lobular fat and the development of type 2 diabetes in overweight or obese patients

**DOI:** 10.3389/fnut.2024.1421032

**Published:** 2024-07-03

**Authors:** Lihui Wang, Yinghao Li, Renfeng Li, Jinwen Luan, Kaiming Cao, Tiancheng Liu, Haiyang Hu, Shanshan Chen, Le Bu, Longhua Liu, Hongzhi Wang, Qing Lu

**Affiliations:** ^1^Department of Radiology, Shanghai East Hospital, Tongji University, Shanghai, China; ^2^Physics Department & Shanghai Key Laboratory of Magnetic Resonance, School of Physics and Electronic Science, East China Normal University, Shanghai, China; ^3^School of Exercise and Health, Shanghai University of Sport, Shanghai, China; ^4^College of Medical Imaging, Shanghai University of Medicine and Health Science, Shanghai, China; ^5^Department of Endocrinology and Metabolism, Shanghai Tenth People's Hospital, Medicine School of Tongji University, Shanghai, China

**Keywords:** obesity, T2DM, mDixon MRI, pancreas fat quantification, intra-lobular fat, inter-lobular fat

## Abstract

Pancreatic fat is associated with obesity and type 2 diabetes mellitus (T2DM); however, the relationship between different types of pancreatic fat and diabetes status remains unclear. Therefore, we aimed to determine the potential of different types of pancreatic fat accumulation as a risk factor for T2DM in overweight or obese patients. In total, 104 overweight or obese patients were recruited from January 2020 to December 2022. The patients were divided into three groups: normal glucose tolerance (NGT), impaired fasting glucose or glucose tolerance (IFG/IGT), and T2DM. mDixon magnetic resonance imaging (MRI) was used to detect pancreatic fat in all three groups of patients. The pancreatic head fat (PHF), body fat (PBF), and tail fat (PTF) in the IFG/IGT group were 21, 20, and 31% more than those in the NGT group, respectively. PHF, PBF, and PTF were positively associated with glucose metabolic dysfunction markers in the NGT group, and inter-lobular fat volume (IFV) was positively associated with these markers in the IFG/IGT group. The areas under the receiver operating characteristic curves for PHF, PBF, and PTF (used to evaluate their diagnostic potential for glucose metabolic dysfunction) were 0.73, 0.73, and 0.78, respectively, while those for total pancreatic volume (TPV), pancreatic parenchymal volume, IFV, and IFV/TPV were 0.67, 0.67, 0.66, and 0.66, respectively. These results indicate that intra-lobular pancreatic fat, including PHF, PTF, and PBF, may be a potential independent risk factor for the development of T2DM. Additionally, IFV exacerbates glucose metabolic dysfunction. Intra-lobular pancreatic fat indices were better than IFV for the diagnosis of glucose metabolic dysfunction.

## 1 Introduction

Worldwide, over 1.9 billion adults are overweight, of which more than 650 million are obese, based on data from the World Health Organization. Obesity, characterized by excessive fat accumulation, is a strong risk factor for many metabolic diseases such as type 2 diabetes mellitus (T2DM), hypertension, and cardiovascular diseases (1, 2). Both hyperplasia (adipogenesis) and hypertrophy contribute to excessive fat accumulation in adipose tissue (3). As a key nutrition-responsive metabolic tissue, adipose tissue stores lipids and secretes different adipokines to regulate whole-body lipid and glucose metabolism, which are strongly associated with metabolic diseases (4–6). Healthy or functional adipose tissue helps to protect from metabolic dysfunctions, while dysfunctional adipose tissue may exacerbate it by different mechanism such as lipotoxicity and secreted proinflammatory adipokines (7).

Adipose tissue is mainly divided into white adipose tissue (WAT) and brown adipose tissue (BAT). WAT can be located in different regions, including subcutaneous white adipose tissue (sWAT), visceral white adipose tissue (vWAT) and other locations including the bone marrow, underline the skin and surrounding the heart (7). Compared with sWAT, the fat mass of vWAT has a stronger positive correlation with the occurrence of metabolic diseases, including T2DM (8, 9). During overweight and obesity, WAT could be highly remodeled including increased adipocyte size, changed secret pattern, cell composition and induced hypoxia and inflammation within adipose tissue (7, 10). Hypoxia within the adipose tissue induced by decreasing adipose tissue blood flow and vascular rarefaction might result in adipocyte death and further macrophage infiltration as well as endothelial dysfunction (10). In addition to enlarged adipose tissue, ectopic fat can also infiltrate other metabolic organs, such as the liver and pancreas, resulting in fatty liver and pancreas disease, respectively (11). Fatty liver disease is strongly associated with insulin resistance and T2DM (12). Lifestyle interventions or certain drug treatments for T2DM, such as thiazolidinediones, can also alleviate fatty liver disease, and vice versa (13). Hypercaloric diets, with high saturated fat and glucose levels, can gradually overburden adipocytes with excessive lipolysis, resulting in an elevated flow of free fatty acids and triglyceride synthesis in the liver (13, 14). Lipid intermediates, such as diacylglycerols, could inhibit insulin signaling pathways by impairing insulin receptor substrate 2 (IRS2) phosphorylation as well as elevating the PKC pathway in the liver resulting in insulin resistance (15). Although the role of fatty liver disease in T2DM has been well studied, the potential relationship between fatty pancreas disease and glucose metabolism has not, despite growing interest.

The pancreas contains two distinct components: the exocrine pancreas, including acinar and ductal cells; and the endocrine pancreas, including islets of Langerhans (16). The exocrine pancreas plays a critical role in digesting carbohydrates, fats, and proteins by secreting digestive enzymes, such as amylases, lipases, and proteinases; while the endocrine pancreas regulates glucose homeostasis by secreting insulin (β cells), glucagon (λ cells), etc. (17, 18). Insulin plays a central role in the downregulation of blood glucose, whereas glucagon upregulates blood glucose. Pancreatic fat has been shown to associations with many different factors such as metabolic syndromes, metabolic dysfunction-associated steatotic liver disease (MASLD) and age etc. (18). Ectopic fat accumulation in the pancreas is associated with obesity and related metabolic diseases such as T2DM (19). Increased body mass index (BMI) and obesity are positively associated with fatty pancreas independent of sex and age, and pancreatic fat is negatively associated with insulin secretion (20). Ectopic fat can be deposited in different parts of the pancreas, including the islets of Langerhans, acinar cells, and inter-lobular stroma (21). Intra-lobular fat in endocrine cells (such as β cells) and acinar cells was shown to contribute to the occurrence of T2DM; hence, decreased intra-lobular fat may help alleviate T2DM (22–24). Inter-lobular fat is usually present during acute pancreatitis; however, it may also be involved in the development of T2DM by stimulating inflammation (25). Both oxidative cell stress and proinflammatory adipokines (such as leptin, adipsin, and resistin) can stimulate local inflammation and further insulin resistance, T2DM and related atherosclerosis (6, 26–28). However, it remains to be determined whether there are similar or different associations between pancreatic intra-, inter-lobular fat and the development of T2DM.

Many methods have been developed to detect fat accumulation in the pancreas, such as: histology, ultrasonography, computed tomography (CT), and magnetic resonance imaging (MRI) (18). While histology is not practical in routine hospital investigations, due to its invasiveness, non-invasive methods are important for pancreatic fat quantification. Ultrasonography is relatively cheap and widely available; however, it has limited use due to unestablished objective standards and unsatisfactory sensitivity and specificity due to investigator bias (29). CT is relatively expensive, requires the intravenous injection of radioactive tracers, and involves radioactive exposure, making it unsuitable for longitudinal fat quantification studies (30). Hence, MRI is commonly used for fat quantification because it does not involve the risk of ionizing radiation and has the advantages of high soft tissue resolution, multiparameter and multisequence scanning, and high repeatability. Multi-echo Dixon (mDixon MRI), a novel MRI technology that enables reliable and highly reproducible pancreatic fat quantification, is widely used (31). Similar to regular magnetic resonance spectroscopy (MRS) and histology, the measurement of proton density fat fraction (PDFF) using mDixon MRI is very accurate and reproducible and is also considered the gold standard (32). Its overall correlation coefficient was 0.999 for repeated scans at different locations, field strengths, and vendors (32). Using advanced mDixon MRI, some studies have documented that elevated BMI, age, and metabolic syndrome are positively associated with pancreatic fat accumulation (18). However, whether pancreatic fat accumulation is an independent risk factor for the development of T2DM has not been comprehensively studied. Therefore, in this study we aimed to determine the potential of different types of pancreatic fat accumulation as a risk factor for T2DM in overweight or obese patients.

## 2 Methods

### 2.1 Participants

**Figure 1:**
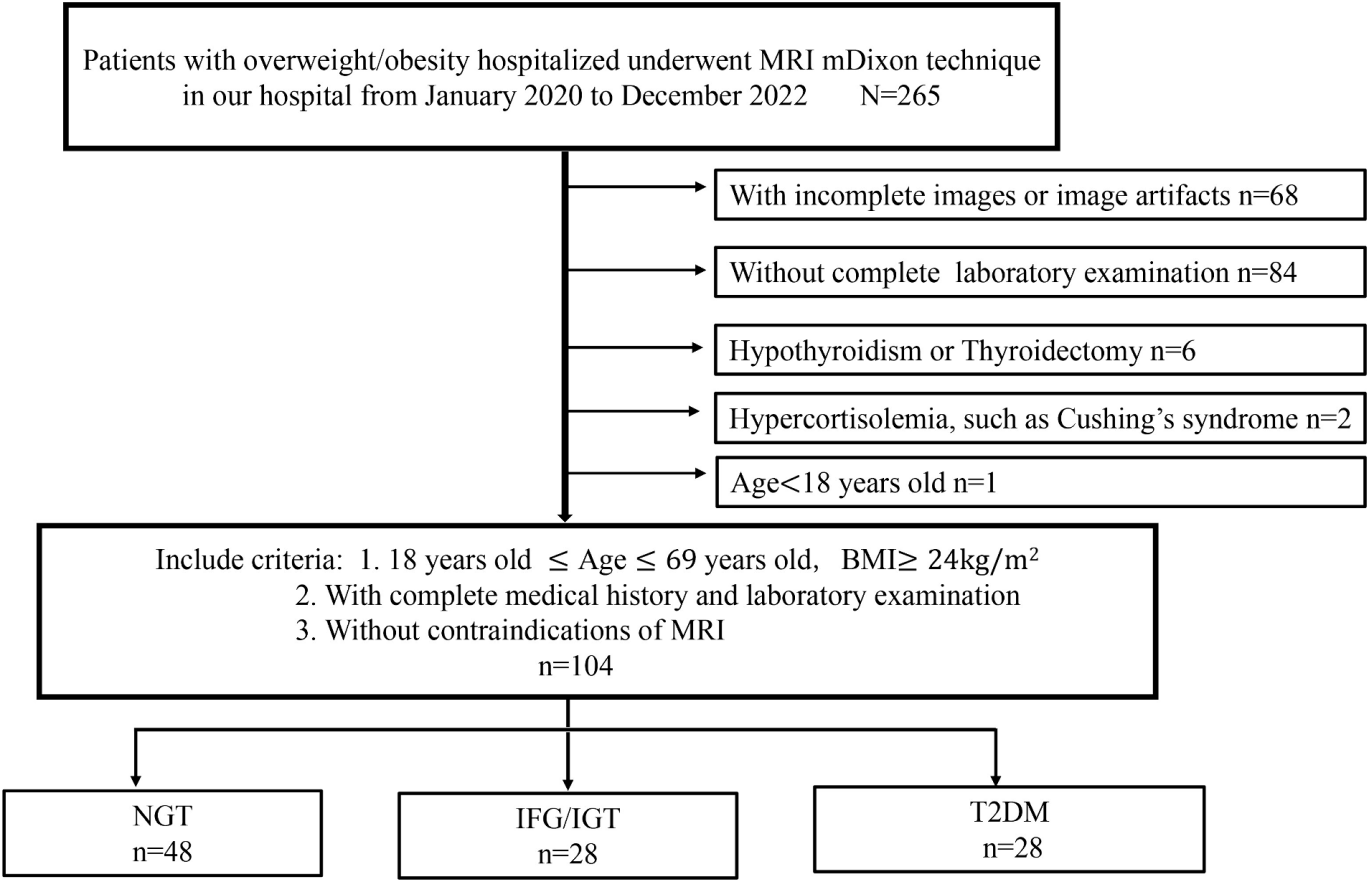
Flow chart of participant selection for this study. A total of 265 hospitalized overweight or obese patients who underwent MRI using the mDixon technique were initially recruited for this study. Sixty eight patients are with incomplete images or image artifacts, 84 patients are without complete laboratory examination, six patients are hypothyroidism or thyroidectomy, two patients are hypercortisolemia and one is under 18 years old. Finally, 104 patients were included in this study and divided into three groups: NGT (*n* = 48), IFG/IGT (*n* = 28), T2DM (*n* = 28).

A total of 265 hospitalized overweight or obese patients who underwent MRI using the mDixon technique were initially recruited for this cross-sectional study. All participants were enrolled in three individual cohort studies conducted at our hospital from January 2020 to December 2022 (Figure 1). The inclusion criteria were: age between 18 and 69 years, a BMI of 24 kg/m^2^ or more, complete medical history and laboratory examination, and no contraindications for MRI. The exclusion criteria were: any form of pancreatic disease, including inflammation, tumor, or autoimmune disease; other endocrine and metabolic diseases, such as type 1 diabetes, hypothyroidism, Cushing’s syndrome, and pituitary dysfunction; incomplete images or image artifacts; and absence of a complete laboratory examination. Based on these criteria, 104 patients were included in the study (Figure 1).

### 2.2 Anthropometry and metabolite assays

The heights and weights of all participants were measured and recorded using standardized and calibrated scales and stadiometers. BMI was calculated as body weight (kg) divided by height squared (m^2^). Waist circumference (WC) was measured midway between the lowest rib and iliac crest in the horizontal plane. Hip circumference (HC) was measured at the level of maximum hip extension. The waist-hip circumference ratio (WHR) was calculated as WC divided by HC.

### 2.3 Biochemical examination

All participants underwent routine biochemical tests after fasting overnight for at least 10 h. These tests included: liver function tests [alanine transaminase (ALT), aspartate transaminase (AST), and gamma glutamyl-transferase (GGT)], lipid panel tests [total cholesterol (TC), triglyceride (TG), high-density lipoprotein cholesterol (HDL-c), low-density lipoprotein cholesterol (LDL-c), lipoprotein a (LPa), apolipoprotein E (ApoE), apolipoprotein B (ApoB), apolipoprotein A (ApoA), and free fatty acid (FFA)], inflammatory factors and amylase [white blood count (WBC), C-reactive protein (CRP), pancreatic amylase, interleukin (IL)-1β, IL-6, IL-8, IL-10, and tissue necrosis factor (TNF)-α], thyroid function tests [thyroid-stimulating hormone (TSH), free T3 (FT3), and free T4 (FT4)], and adrenocortical function and uric acid (UA) (cortisol, UA).

### 2.4 Oral glucose tolerance test and calculations

All participants underwent a 75 g oral glucose tolerance test (OGTT) after overnight fasting. Venous blood samples were drawn at 0, 30, 60, and 120 min to measure the plasma glucose, serum insulin, and C-peptide concentrations. Fasting blood glucose (FBG) levels were measured using the glucose oxidase method, and serum insulin and C-peptide levels were estimated using the chemiluminescence method. Hemoglobin A1c (HbA1c) levels were measured using high-performance liquid chromatography.

The calculation formulas for clinical assessment of insulin sensitivity and β-cell function were as follows: homeostasis model assessment of insulin resistance (HOMA-IR) = FBG [mg/dL] × fasting insulin (FINS) [μIU/mL]/22.5; Matsuda index = 10,000/(FBG [mg/dL] × FINS [μIU/mL] × mean glucose [mg/dL] × mean insulin [μIU/mL])^1/2^; homeostasis model assessment of beta-cell function (HOMA-β) = 20 × FINS [μIU/mL]/(FBG-3.5) [mg/dL]; insulinogenic index (IGI) = Δ insulin (INS)0–30/Δ glucose (GLU) 0–30 = (INS30 - INS0) [μIU/mL]/ (GLU30 - GLU0) [mg/dL], where INSx and GLUx represent insulin and glucose values at time x min during the OGTT, respectively; Area under the curve of plasma insulin (AUCINS) 0 ~ 120 [min*μIU/mL]/area under the curve of plasma glucose (AUCGLU) 0 ~ 120 [min*mg/dL], wherein AUCGLU 0 ~ 120 and AUCINS 0 ~ 120, which were calculated using the trapezoid rule, represented the areas under the curve of plasma glucose and insulin secretion rates during the OGTT from 0 to 120 min, respectively; and oral Disposition Index (oDI) = IGI/FINS.

### 2.5 Separation and quantification of pancreatic fat, vWAT, and sWAT

A 3.0 T MRI system (Ingenia, Philips Healthcare, Best, Netherlands) was used for the MRI examinations. Abdominal MRI examinations for all participants were performed after at least 10 h of fasting in the supine position. The modified Dixon (mDixon MRI) Quant sequence was applied for the quantitative assessment of visceral fat disposition in all three groups of patients: normal glucose tolerance (NGT), impaired fasting glucose or glucose tolerance (IFG/IGT), and T2DM. This modified 6- echoes Dixon sequence included a repetition time of 15 ms, 6 echoes time = TE1/ΔTE 1.15 ms/1.15 ms, flip angle of 3°, matrix size of 188 × 155, field of view of 320 mm, and slice thickness of 3 mm (33). Three-dimensional axial images were captured from the diaphragmatic dome to the pelvic floor during a single breath-hold. After the acquisition, multislice two-dimensional axial images were reconstructed, and five maps, including water, fat, fat fraction, R2*, and T2*, were automatically generated. Using a post-processing workstation (Philips Intellispace; Philips Healthcare, Best, The Netherlands), pancreatic PDFF was measured in the pancreatic intralobular fat by manually placing an approximate ROI of 1 cm^2^ within the head, body, and tail of the pancreas (34). The ROI sizes were selected according to the total extent of the organ or tissue of interest (34). All ROIs were placed to avoid major vessels, ducts, collection systems, and imaging artifacts (35). The pancreatic head, body, and tail diameters were determined as previously described (36). The cross-sectional area of sWAT and vWAT were quantified using the generated fat maps mentioned above. The two-dimensional fat area was multiplied by the slice thickness, followed by all slice levels added to calculate the volumes of sWAT and vWAT.

**Figure 2:**
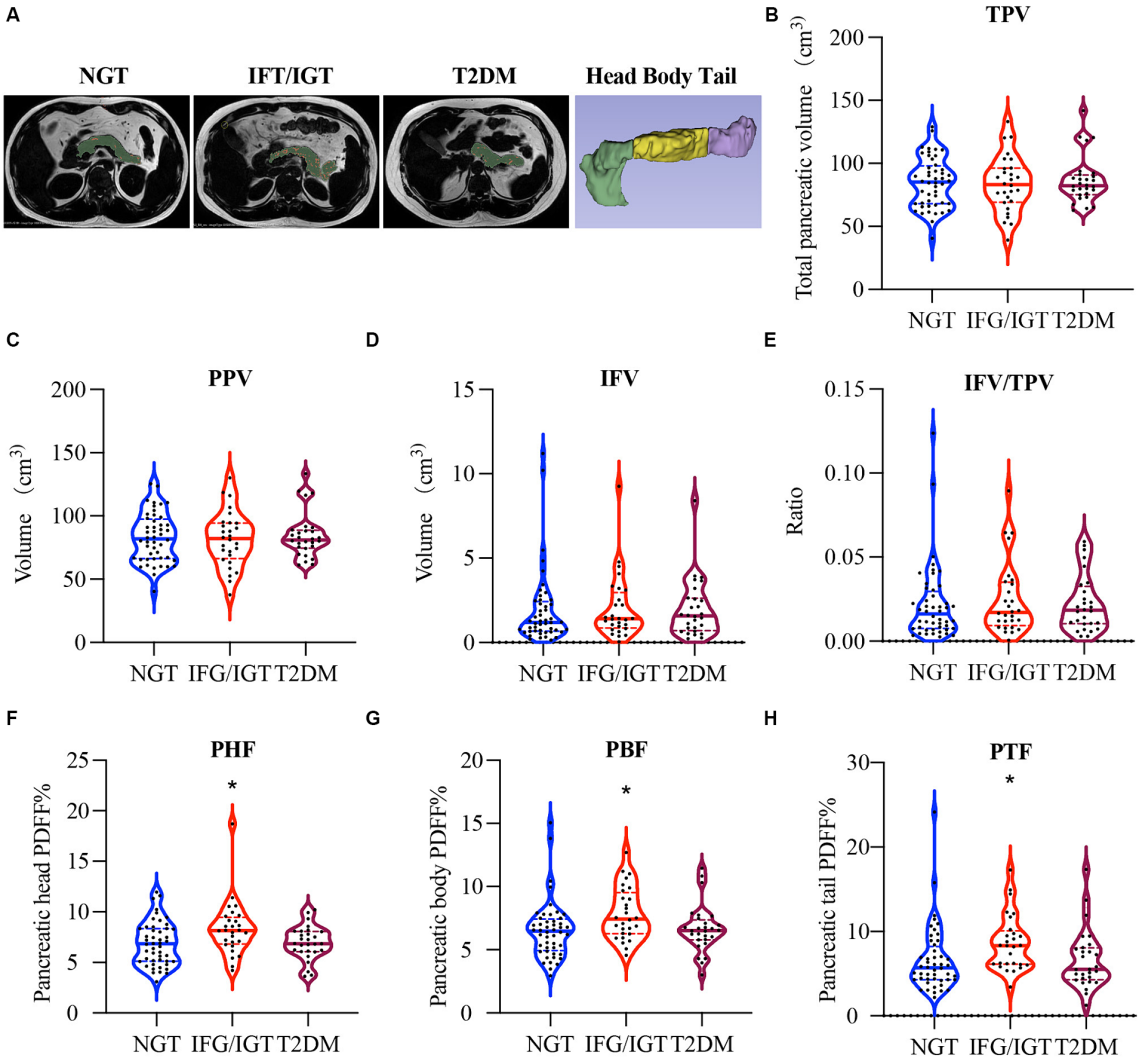
More pancreatic fat in IFG/IGT than NGT patients. **(A)** Representative images of intra-lobular fat, including pancreas head, body, and tail dissection and inter-lobular fat in participants with different glucose metabolic statuses. Pancreatic parenchyma including intra-lobular fat is shown in green, inter-lobular pancreatic fat is shown in red. **(B–H)** Violin plots for **(B)** total pancreas volume (TPV), **(C)** pancreatic parenchymal volume (PPV), **(D)** inter-lobular fat volume (IFV), **(E)** the ratio of inter-lobular fat volume to total pancreatic volume (IFV/TPV), **(F)** pancreatic head fat (PHF), **(G)** pancreatic body fat (PBF), and **(H)** pancreatic tail fat (PTF). NGT, normal glucose tolerance; IFG/IGT, impaired fasting glucose or glucose tolerance; T2DM, type 2 diabetes mellitus. *p < 0.05, one way ANOVA.

The inter-lobular fat in the pancreatic region was quantified using mDixon method and U-Net deep learning network model based on previously established methods (37–39). Generally, a grade-connected U-Net deep learning network model was used for pancreatic fat segmentation (38). The U-Net network is constructed based on Fully Convolutional Network with encoder-decoder structure (39). The encoder part is sampled by several convolution-pooling processes of the input image, while the decoder section uses dehumination and feature stitching to achieve a more feature layer of the field of vision, improves the network’s attention to edge features, and improves the segmentation performance of the model. This study uses the grade-connected nnU-Net network with automated image pre-processing and training parameters. In the image pre-processing stage, it automatically sets up cutting, re-sampling, rotation and other standardized processes according to sample information. In the training, we use learning rate attenuation to improve the network learning ability. During the post-processing process, keep the maximum connected domain to generate dividing images based on prior knowledge. In fat-fraction images, inter-lobular fat showed a high-signal-intensity area in the pancreas, comparable to subcutaneous fat. After preprocessing with denoising, enhancement, and image calibration, the image was divided into two areas using a threshold method: fat and non-fat areas. During the postprocessing stage, isolated noise points, filled holes, and connected broken areas were used to obtain more accurate segmentation results. Segmentation of pancreatic inter-lobular fat was performed using a threshold of 60% signal intensity (36). Representative images show the whole pancreas, the inter-lobular fat, and also dissect the pancreatic intra-lobular fat, including three different parts (head, body, and tail) as: total pancreatic volume (TPV), pancreatic parenchymal volume (PPV), inter-lobular fat volume (IFV), the ratio of inter-lobular fat volume to total pancreatic volume (IFV/TPV), pancreatic head fat (PHF), pancreatic body fat (PBF), and pancreatic tail fat (PTF) (Figure 2A).

### 2.6 Statistical analysis

SPSS version 25.0 (SPSS Inc., Chicago, IL, United States) was used for all statistical analyses. Normal distribution of data was tested using the Kolmogorov–Smirnov test. For the analysis of seven different pancreatic indexes in three groups (NGT, IFG/IGT, T2DM), data are presented as means ± standard deviations, and significant analysis was based on the one-way ANOVA. Correlation studies in all three models (unadjusted, Model 1, and Model 2) were performed using Spearman’s correlation coefficients. To avoid the confounding effects of age, BMI, and WHR, adjusted Model 1 was applied to study the correlation of pancreatic indices with glucose metabolism, inflammation, and liver function indices in the three groups. Furthermore, to avoid masking by liver dysfunction, Model 2 was adjusted for age, BMI, WHR, ALT, AST, and GGT.

## 3 Results

### 3.1 Clinical characteristics of the patient groups

To investigate the potential roles of pancreatic fat in T2DM, 104 overweight/obese patients were divided into three groups: 48 patients in the NGT group, 28 in the IFG/IGT group, and 28 in the T2DM group based on the WHO-recommended criteria (40). There were no significant differences in age, BMI, WHR, sWAT, or vWAT among the three groups (Table 1). Regarding the glucose metabolism index, oDI, IGI, and HOMA-β were gradually decreased during the developing of diabetes; while HbA1c, GA, PBG at 0.5, 1, 2, and 3 h were significantly increased for both IFG/IGT and T2DM groups compared with the NGT group. However, the insulin levels at the 2, and 3 h time points were higher in the IFG/IGT group than in the NGT group, but much lower in the T2DM group than in the NGT group at 0.5 h and 1 h time points. Regarding inflammation status, no significant differences existed among the groups for IL-10, IL-8, IL6, IL-1β, TNF-α, and CRP. Furthermore, no significant changes in lipid metabolism were observed for TG, TC, LDL-c, HDL-c, ApoA, ApoB, or ApoE among the groups. However, the LPa was lower in the T2DM group than in the NGT group. Liver dysfunction worsened during the course of diabetes, as indicated by GGT, AST, and ALT levels. There were no significant differences in ACTH, UA, TSH, FT3, and FT4 levels among the three groups (Table 1).

**Table 1:** Clinical characteristics for NGT, IFG/IGT, and T2DM patients.

	Total *n* = 104	NGT *n* = 48	IFG/IGT *n* = 28	T2DM *n* = 28	*p* value
Gender (male, female)	(57, 37)	(31, 17)	(14, 14)	(22, 6)	0.0826
Age, yrs	36.49 ± 11.62	34.23 ± 11.27	39.04 ± 10.32	37.82 ± 13.03	0.172
BMI, kg/m^2^	33.45 ± 5.89	34.47 ± 7.11	32.84 ± 3.6	32.3 ± 5.25	0.505
WHR	0.95 ± 0.1	0.96 ± 0.1	0.94 ± 0.1	0.97 ± 0.1	0.691
sWAT, cm^3^	4011.75 ± 1903.39	4275.05 ± 2249.22	3975.94 ± 1446.23	3596.18 ± 1618.35	0.326
vWAT, cm^3^	2628.31 ± 1165.61	2525.07 ± 1246.15	2529.52 ± 1182.37	2904.07 ± 986.84	0.346
Glucose metabolism index					
oDI	0.08 ± 0.07	0.12 ± 0.08	0.08 ± 0.06*	0.03 ± 0.03^###^	<0.001
IGI	1.16 ± 0.98	1.56 ± 1.03	1.22 ± 0.82	0.42 ± 0.49^###^	<0.001
Matsuda. index	424.9 ± 32.26	430.61 ± 27.03	421.95 ± 35.44	418.07 ± 36.41	0.226
HOMA-β	257.41 ± 234.36	302.9 ± 241.48	270.83 ± 249.21	166.02 ± 182.75^##^	0.045
HOMA-IR	4.58 ± 4.33	3.32 ± 2.55	4.02 ± 2.56	7.28 ± 6.56^##^	0.002
AUCCP/AUCGLU	1.18 ± 0.54	1.43 ± 0.5	1.27 ± 0.36	0.66 ± 0.38^###^	<0.001
AUCINS/AUCGLU	10.8 ± 6.66	12.94 ± 5.84	12.09 ± 6.59	5.85 ± 5.58^###^	<0.001
AUCCP.0–120	1200.63 ± 487.16	1290.01 ± 471.47	1357.94 ± 385.56	887.71 ± 478.56^###^	<0.001
AUCINS.0–120	10968.9 ± 6737.33	11662.12 ± 5747.67	12887.6 ± 7040.84	7861.84 ± 7169.18^#^	0.011
AUCGLU.0–120	1084.89 ± 316.99	896.57 ± 113.64	1066.52 ± 108.83***	1426.1 ± 406.03^###^	<0.001
HbA1C, %	6.17 ± 1.86	5.4 ± 0.34	5.55 ± 0.51	8.19 ± 2.73^###^	<0.001
GA, %	13.88 ± 5.75	11.75 ± 1.48	12.47 ± 1.05*	19.3 ± 9.33^###^	<0.001
CPE (0 h), ng/mL	3.76 ± 1.79	3.69 ± 1.68	4.04 ± 2.05	3.62 ± 1.73	0.646
CPE2 (0.5 h), ng/mL	9.3 ± 4.22	10.98 ± 4.09	9.79 ± 3.24	5.89 ± 3.39^###^	<0.001
CPE3 (1 h), ng/mL	11.78 ± 4.65	13.32 ± 4.01	12.7 ± 3.79	8.16 ± 4.66^###^	<0.001
CPE4 (2 h), ng/mL	12.11 ± 4.57	11.81 ± 4.13	14.4 ± 4.22*	10.33 ± 4.81	0.003
CPE5 (3 h), ng/mL	8.52 ± 3.72	7.24 ± 3.41	10.05 ± 4.14**	9.16 ± 3.07^#^	0.004
FINS, μIU/mL	18.22 ± 15.23	15.6 ± 11.73	17.87 ± 11.12	23.05 ± 22.04	0.120
0.5 h PINS, μIU/mL	93.9 ± 63.74	112.56 ± 61.2	101.3 ± 65.21	53.74 ± 48.76^###^	<0.001
1 h PINS, μIU/mL	111.97 ± 69.14	125.44 ± 61.05	126.07 ± 71.77	73.89 ± 67.66^##^	0.003
2 h PINS, μIU/mL	99.71 ± 73.83	86.49 ± 54.47	130.24 ± 79.07*	91.85 ± 89.63	0.034
3 h PINS, μIU/mL	50.54 ± 49.62	32.15 ± 30.03	65.7 ± 57.16**	66.83 ± 58.67^##^	<0.001
FBG, mmol/L	5.48 ± 1.93	4.74 ± 0.55	4.96 ± 0.79	7.26 ± 2.91^###^	<0.001
0.5 h PBG, mmol/L	9.01 ± 2.11	8.21 ± 1.3	8.94 ± 1.28*	10.5 ± 3.03^###^	<0.001
1 h PBG, mmol/L	10.25 ± 3.06	8.52 ± 1.63	10.19 ± 1.82***	13.4 ± 3.54^###^	<0.001
2 h PBG, mmol/L	9.27 ± 4.34	6.53 ± 0.78	8.84 ± 0.99***	14.41 ± 5.36^###^	<0.001
3 h PBG, mmol/L	6.76 ± 3.85	4.84 ± 1.05	5.97 ± 1.55**	10.99 ± 5.26^###^	<0.001
Inflammation factors					
IL-10	5.06 ± 0.34	5.07 ± 0.33	5.1 ± 0.5	5 ± 0.01	0.591
IL-8	89.82 ± 103.12	91.03 ± 116.52	93.72 ± 97.05	83.99 ± 89.34	0.945
IL-6	3.48 ± 1.67	3.82 ± 2.07	2.81 ± 0.95*	3.63 ± 1.35	0.059
IL-1β	6.87 ± 4.28	6.76 ± 4.35	6.5 ± 3.24	7.43 ± 5.13	0.743
TNF-α	7.88 ± 3.83	7.83 ± 4.82	7.58 ± 3.51	8.24 ± 2.04	0.834
CRP	2.75 ± 3.65	2.92 ± 4.01	1.95 ± 3.06	3.22 ± 3.51	0.471
Lipid metabolism index					
LPa	168.71 ± 264.29	193.61 ± 201.23	186.51 ± 411.94	107.15 ± 133.68^#^	0.385
ApoE	17.76 ± 24.48	16.67 ± 22.18	9.71 ± 12.37	27.99 ± 33.45	0.095
ApoB	1.09 ± 0.24	1.06 ± 0.25	1.11 ± 0.24	1.11 ± 0.23	0.635
ApoA	1.21 ± 0.19	1.2 ± 0.19	1.26 ± 0.18	1.16 ± 0.2	0.193
TG	2.2 ± 1.51	1.98 ± 1.43	2.05 ± 1.02	2.75 ± 1.92	0.082
FFA	0.61 ± 0.25	0.54 ± 0.24	0.64 ± 0.27	0.69 ± 0.24^#^	0.034
CHOL	5.16 ± 1.12	5.08 ± 1.11	5.12 ± 0.89	5.32 ± 1.36	0.670
LDL-C	3.2 ± 0.83	3.14 ± 0.85	3.17 ± 0.81	3.32 ± 0.83	0.641
HDL-C	1.1 ± 0.21	1.11 ± 0.23	1.13 ± 0.19	1.05 ± 0.21	0.352
Liver function index					
GGT	50.07 ± 50.73	41.27 ± 35.45	52.04 ± 67.87	63.67 ± 51.99	0.181
AST	31.25 ± 26.28	24.73 ± 20.36	26.54 ± 11.27	47.74 ± 37.99^##^	0.004
ALT	48.85 ± 59.95	35.75 ± 38.13	39.25 ± 30.67	82.11 ± 94.5^#^	0.009
Others					
Cortisol	278.39 ± 106.68	293.24 ± 106.47	249.45 ± 111.51	284.41 ± 100.11	0.266
ACTH	33.09 ± 17.6	33.57 ± 19.3	26.73 ± 11.15	37.95 ± 18.3	0.080
UA	437.25 ± 121.87	437.34 ± 136.06	421.25 ± 114.44	454.31 ± 103.02	0.614
TSH	2.38 ± 1.22	2.34 ± 1.07	2.38 ± 1.07	2.45 ± 1.62	0.940
FT3	5.19 ± 0.72	5.28 ± 0.7	5.04 ± 0.64	5.2 ± 0.84	0.419
FT4	15.15 ± 3.14	15.38 ± 3.33	14.97 ± 3.02	14.94 ± 3.05	0.817

Data were presented as mean ± SD, *p < 0.05, **p < 0.01, ***p < 0.001 in the IFG/IGT vs NGT group; ^#^*p* < 0.05, ^##^*p* < 0.01, ^###^*p* < 0.001 in the T2DM vs NGT group based on one-way ANOVA after Bonferroni correction.

NGT, normal glucose tolerance; IFG/IGT, impaired fasting glucose or glucose tolerance; T2DM, type 2 diabetes; WHR, Waist-hip circumference ratio; sWAT, subcutaneous white adipose tissue; vWAT, visceral white adipose tissue; oDI, oral Disposition Index; IGI, insulinogenic index; HOMA-β, homeostasis model assessment-β; HOMA-IR, homeostasis model assessment of insulin resistance; AUCCP/AUCGLU, Area under curve of C peptide/ area under curve of glucose; AUCINS/AUCGLU, Area under curve of insulin/ area under curve of glucose; AUCCP.0–120, area under curve of C peptide 0–120 min; AUCINS.0–120, area under curve of insulin 0–120 min; AUCGLU.0-120:area under curve of glucose 0–120 min; GA, glycated albumin; CPE:C peptide estimation; FINS, fasting insulin; PINS, plasma insulin; FBG, fasting blood glucose; PBG, plasma blood glucose; IL-10, interleukin-10; TNF-α, tumor necrosis factor-α; CRP, C-reactive protein; LPa, lipoprotein a; ApoE, apolipoprotein E; ApoB, apolipoprotein B; ApoA, apolipoprotein A; TG, triglyceride; FFA, free fatty acid; CHOL, cholesterol; LDL-c, low-density lipoprotein cholesterol; HDL-c, high-density lipoprotein cholesterol; GGT, gamma glutamyltranspeptidase; AST, aspartate aminotransferase; ALT, alanine aminotransferase; ACTH, adrenocorticotropic hormone; UA, uric acid; TSH, thyroid stimulating hormone; FT3, free triiodothyronine; FT4, free thyroxine.

### 3.2 More pancreatic fat in patients with IFG/IGT than NGT

There were no significant differences in TPV (Figure 2B), PPV (Figure 2C), IFV (Figure 2D), and IFV/TPV (Figure 2E) among all three groups of patients. Interestingly, the PHF in the IFG/IGT group was 21% higher than that in the NGT group, whereas the PHF in the T2DM group was comparable to that in the NGT group (Figure 2F). Similarly, PBF and PTF in the IFG/IGT group were 20 and 31% more than those in the NGT group, respectively; whereas there were no significant differences between the NGT and T2DM group (Figures 2G,H).

### 3.3 Distinct correlation between pancreatic fat and metabolic indexes

**Figure 3:**
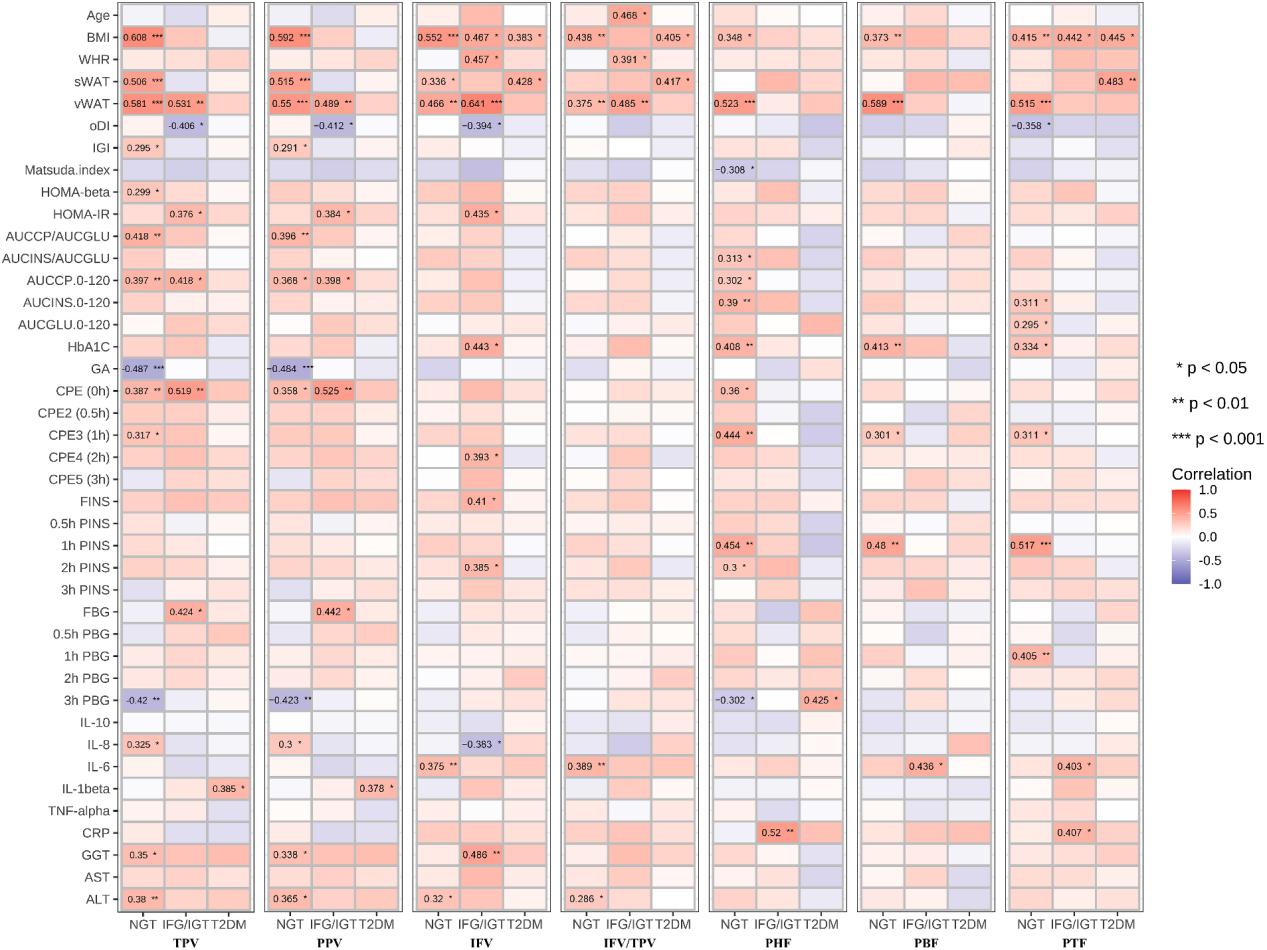
Correlation map between all seven pancreatic indexes with clinical indexes in the three groups. The seven pancreatic indexes are including TPV, PPV, IFV, IFV/TPV, PHF, PBF, and PTF. The clinical indexes are including Age, BMI, WHR, sWAT, vWAT, oDI, IGI, Matsuda. Index, HOMA-beta, HOMA-IR, AUCCP/AUCGLU, AUCINS/AUCGLU, AUCCP.0–120, AUCINS 0–120, AUCGLU0-120, HbA1C, GA, CPE (0 h), CPE2 (0.5 h), CPE3 (1 h), CPE4 (2 h), CPE5 (3 h), FINS, 0.5 h FINS, 1 h FINS, 2 h FINS, 3 h FINS, FBG, 0.5 h FBG, 1 h FBG, 2 h FBG, 3 h FBG, IL-10, IL-8, IL-6, IL-1beta, TNF-alpha, CRP, GGT, AST, ALT. These three groups are NGT, IFG/IGT, T2DM. *p < 0.05, **p < 0.01, ***p < 0.001, *t*-test.

To study which parts of pancreatic fat are severe risk factors for T2DM, all seven pancreatic indices including TPV, PPV, IFV, IFV/TPV, PHF, PBF, and PTF were correlated with the anthropometric, glucose metabolism, inflammation, and liver function indexes in the three groups (Figure 3). We found that age was positively associated with IFV/TPV in the IFG/IGT group; however, there was no significant association with other pancreatic indices. BMI and vWAT were strongly and positively correlated with all pancreatic indices in most combinations, particularly in the NGT and IFG/IGT groups. For the glucose metabolism index, both TPV and PPV were positively associated with glucose metabolic dysfunction markers such as HOMA-β, HOMA-IR, AUCCP/AUCGLU, AUCCP.0–120, and C-peptide estimation (CPE) (0 h) in the NGT and IFG/IGT groups, but not in the T2DM group. Intriguingly, there was a positive association between IFV and glucose metabolic dysfunction markers such as HOMA-IR, HbA1C, CPE4 (2 h), FINS, and 2 h FINS in the IFG/IGT group, but not in the NGT or T2DM groups. In contrast, PHF, PBF, and PTF were significantly positively associated with glucose metabolic dysfunction markers, including HbA1c, CPE4 (1 h), and 1 h PINS in the NGT group but not in the IFG/IGT or T2DM groups (Figure 3). Furthermore, PHF, PBF, and PTF were positively associated with inflammation marker such as IL-6 and CRP in the IFG/IGT group (Figure 3). It is worth noting that TPV, PPV, and IFV were positively associated with liver dysfunction markers including GGT and ALT especially in the NGT group, which may cofound these associations.

### 3.4 Inter-lobular fat exacerbated glucose metabolic dysfunction

**Figure 4:**
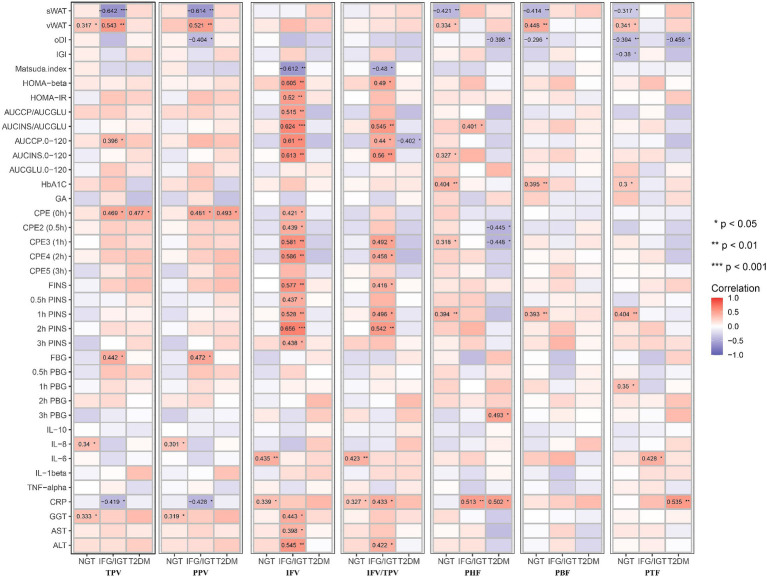
Correlation map between all seven pancreatic indexes with clinical indexes in the three groups after adjusting with age, BMI, WHR. These pancreatic indexes, clinical indexes and groups are the same as in Figure 3. BMI, body mass index; WHR, waist-hip ratio. *p < 0.05, **p < 0.01, ***p < 0.001, *t*-test.

To avoid the confounding effects of age, BMI, and WHR, an adjusted Model 1 applied to study the correlation of pancreatic indices with glucose metabolism, inflammation, and liver function indices in the three groups independent of age, BMI and WHR (Figure 4). It shows that PHF, PBF, and PTF were negatively associated with sWAT, but significantly positively associated with vWAT in the NGT group. In addition, IFV and IFV/IPV were positively correlated with glucose metabolic dysfunction markers but negatively associated with the Matsuda index in the IFG/IGT group, which was consistent with the unadjusted model. Similar to the unadjusted model, PHF, PBF, and PTF were positively associated with glucose metabolic dysfunction markers including HbA1c and 1 h PINS in the NGT group; however, no significant association between PFs (PHF, PBF, and PTF) and glucose metabolic dysfunction was observed in both the IFG/IGT and T2DM groups. Both IFV and IFV/IPV were positively associated with the IL-6 and CRP levels in the NGT group. PHF and PTF were also positively correlated with the inflammatory marker CRP in the IFG/IGT and T2DM groups, indicating that inflammatory markers may be risk factors for the development of diabetes (Figure 4). Meanwhile, the liver dysfunction markers GGT, AST, and ALT were significantly positively associated with IFV in the IFG/IGT group, but not in the NGT or T2DM groups, which may indicate a correlation between IFV and glucose metabolic dysfunction markers.

### 3.5 PHF and PBF were glucose metabolism dysfunction markers independent of liver dysfunction

**Figure 5:**
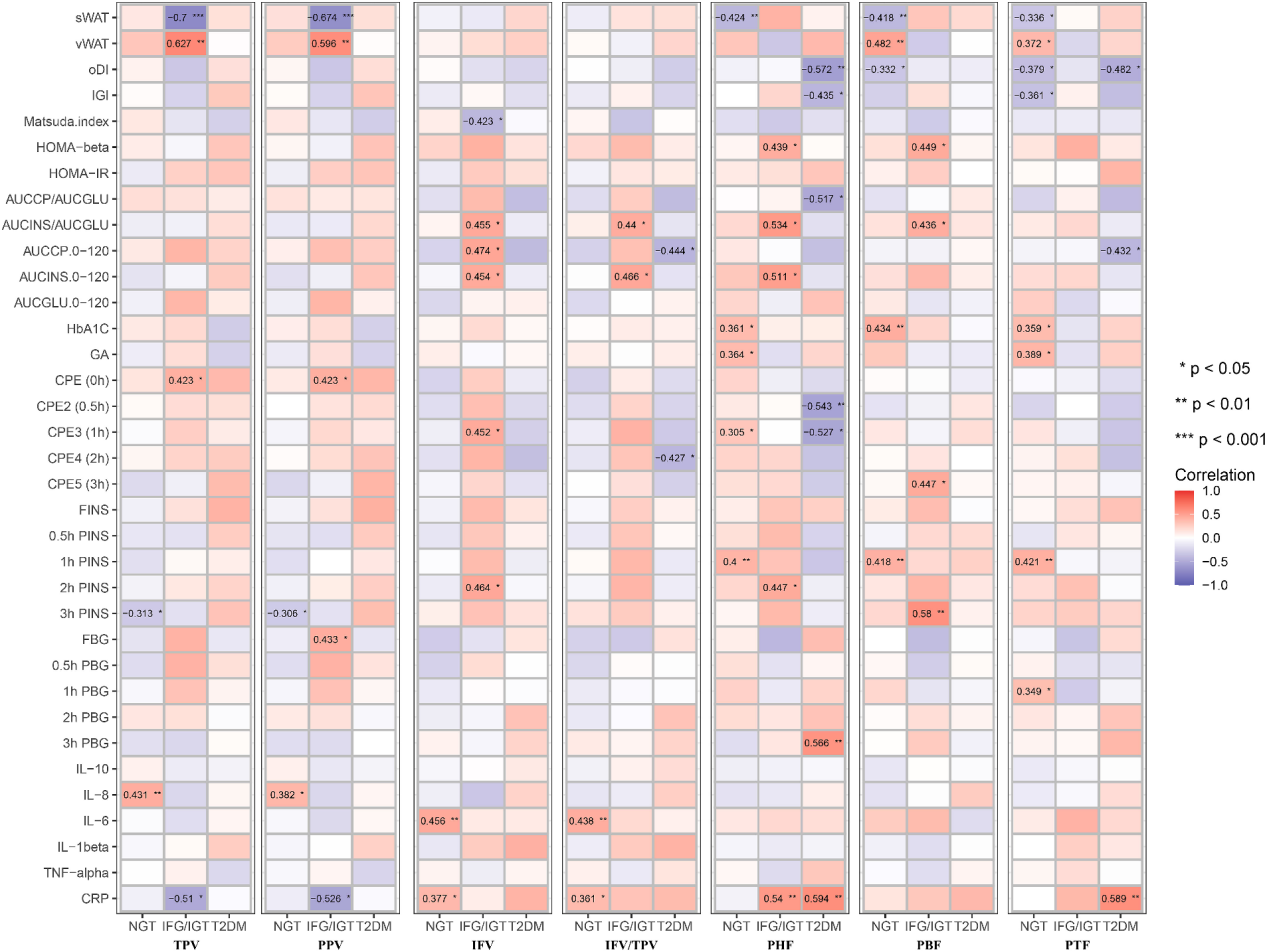
Correlation map between all seven pancreatic indexes with clinical indexes in the three groups after adjusting with age, BMI, WHR, GGT, AST, and ALT. These pancreatic indexes, clinical indexes and groups are the same as in Figure 3. BMI, body mass index; WHR, waist-hip circumference ratio; GGT, gamma glutamyl transpeptidase; AST, aspartate aminotransferase; ALT, alanine aminotransferase. *p < 0.05, **p < 0.01, ***p < 0.001, *t*-test.

To avoid masking by liver dysfunction, Model 2 was adjusted for age, BMI, WHR, ALT, AST, and GGT. Consistent with the unadjusted or adjusted Model 1, PHF, PBF, and PTF were positively associated with glucose metabolic dysfunction markers in the NGT group, whereas PHF and PBF were positively associated with glucose metabolic dysfunction markers in the IFG/IGT group. Consistent with the unadjusted model and Model 1, IFV was also positively associated with glucose metabolic dysfunction markers in the IFG/IGT group in adjusted Model 2. Furthermore, PHF and PTF were negatively associated with oDI and IGI but positively correlated with 3 h PBG in the T2DM group. Interestingly, in Model 2, both PHF and PBF were positively associated with the glucose metabolic dysfunction index including HOMA-β and AUCINS/AUCGLU in IFG/IGT group, which indicated that PHF and PBF accumulation may be independent risk factors for insulin resistance (Figure 5).

### 3.6 Pancreatic intra-lobular fat was a better index than inter-lobular fat for glucose metabolic dysfunction

**Figure 6:**
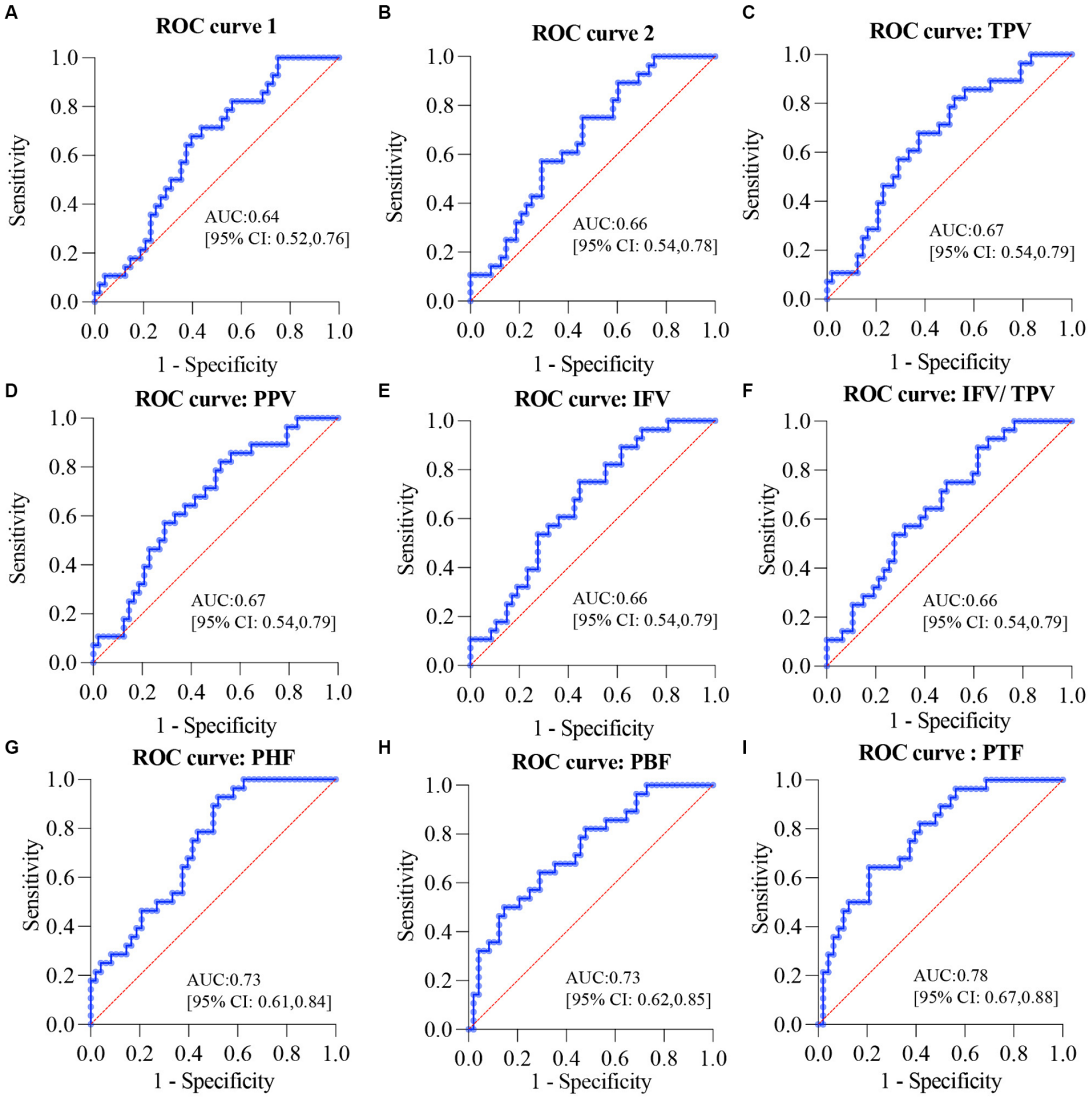
ROC curves for the diagnosis of glucose metabolic dysfunction in overweight or obese patients. **(A)** ROC curve for the combination of age, BMI, and WHR. **(B)** ROC curve for the combination of age, BMI, WHR, ALT, AST, and GGT. **(C)** ROC curve for the combination of TPV, age, BMI, WHR, ALT, AST, and GGT. **(D)** ROC curve for the combination of PPV, age, BMI, WHR, ALT, AST, and GGT. **(E)** ROC curve for the combination of IFV, age, BMI, WHR, ALT, AST, and GGT. **(F)** ROC curve for the combination of IFV/TPV, age, BMI, WHR, ALT, AST, and GGT. **(G)** ROC curve for the combination of PHF, age, BMI, WHR, ALT, AST, and GGT. **(H)** ROC curve for the combination of PBF, age, BMI, WHR, ALT, AST, and GGT. **(I)** ROC curve for the combination of PTF, age, BMI, WHR, ALT, AST, and GGT. ROC, receiver operating characteristic; BMI, body mass index; WHR, waist-hip circumference ratio; GGT, gamma glutamyl transpeptidase; AST, aspartate aminotransferase; ALT, alanine aminotransferase; TPV, total pancreas volume; PPV, pancreatic parenchymal volume; IFV, inter-lobular fat volume; IFV/TPV, the ratio of inter-lobular fat volume to total pancreatic volume; PHF, pancreatic head fat; PBF, pancreatic body fat; PTF, pancreatic tail fat.

To study whether pancreatic indices could be applied for the early diagnosis of glucose metabolic dysfunction, both the NGT and IFG/IGT groups were used logistic regression study and evaluated by using receiver operating characteristic (ROC) curves. The area under the ROC curve (AUC) for the combination of age, BMI, and WHR was 0.64 (*p* = 0.0429) (Figure 6A), and for the combination of age, BMI, WHR, ALT, AST, and GGT was 0.66 (*p* = 0.0189) (Figure 6B), indicating that it was not sufficient to diagnose glucose metabolic dysfunction if only indices including age, BMI, WHR, ALT, AST, and GGT were included. Each of the seven pancreatic indices was combined with age, BMI, WHR, ALT, AST, and GGT to test their potential for the early diagnosis of glucose metabolic dysfunction. TPV, PPV, IFV, and IFV/TPV did not significantly improve the AUC, with values of 0.67 (*p* = 0.0168), 0.67 (*p* = 0.0168), 0.66 (*p* = 0.0175), and 0.66 (*p* = 0.0175), respectively (Figures 6C–F). In contrast, pancreatic intra-lobular fat, including PHF, PBF, and PTF, significantly improved the early diagnosis of glucose metabolic dysfunction, with AUCs of 0.73 (*p* = 0.0011), 0.73 (*p* = 0.0007), and 0.78 (*p* < 0.0001, respectively) (Figures 6G–I). These data indicate that pancreatic intra-lobular fat, including PHF, PBF, and PTF, is a better index for the diagnosis of glucose metabolic dysfunction than inter-lobular fat.

## 4 Discussion

In this study, 104 overweight or obese patients were recruited to investigate the roles of different types of the pancreatic fat in the development of T2DM using mDixon MRI. Glucose metabolism indices could distinguish between the NGT, IFG/IGT, and T2DM groups. PHF, PBF, and PTF levels were significantly higher in the IFG/IGT group than in the NGT group. Furthermore, regarding the correlation between pancreatic fat and the glucose metabolic index, PHF, PBF, and PTF were positively associated with glucose metabolic dysfunction markers in the NGT group in all three correlation models; whereas IFV showed a strong positive correlation with glucose metabolic dysfunction markers in the IFG/IGT group, but not in the NGT group. After adjusting for age, BMI, WHR, and ALT, AST, and GGT levels, both PHF and PBF levels were positively associated with glucose metabolic dysfunction in the NGT and IFG/IGT groups. These results strongly indicate that PHF and PBF may be independent risk factors for early stage T2DM, and that IFV exacerbates glucose metabolic dysfunction. Furthermore, the ROC showed that PHF, PBF, and PTF were better indices than inter-lobular fat in the diagnosis of glucose metabolic dysfunction. To the best of our knowledge, this is the first study to distinguish the distinct roles of PHF, PBF, PTF, and IFV in different stages of T2DM in overweight and obese Asian patients.

mDixon MRI is suitable for quantifying different parts of pancreatic fat in overweight or obese patients. As a noninvasive method for detecting pancreatic fat, ultrasonography is not suitable for distinguishing the different parts of pancreatic fat, including PHF, PBF, PTF, and IFV, especially in overweight or obese patients. Due to ectopic fat, it is difficult to get satisfactory images using ultrasonography (29). Although CT is much more sensitive than ultrasonography, ionizing radiation limits its widespread use for pancreatic fat quantification (41). Recently, mDixon MRI has become widely used for quantifying pancreatic fat because of its reliable reproducibility and safety. Dixon images are highly correlated with CT images in pancreatic fat quantification (41, 42). In this cross-sectional study, mDixon MRI was used to measure different parts of pancreatic fat in overweight and obese patients, saving them from the ionizing radiation of CT scanning, but with similar sensitivity. To maintain the randomness of this study, radiologists were blinded to all other clinical indices of the patients and used mDixon MRI to measure the different parts of the pancreatic fat using a well-established protocol (34). All data were analyzed by two different researchers and the results were no significant difference between two different researchers using paired-*t* test.

Both inter- and intra-lobular fat accumulation in the pancreas disturb normal pancreatic function, including glucose metabolism. The endocrine pancreas plays a critical role in regulating glucose homeostasis mainly by secreting insulin (β cells) and glucagon (λ cells) (17, 18). Ectopic fat accumulation may result in the secretion of proinflammatory adipokines to stimulate inflammation, which exacerbates insulin resistance and related metabolic diseases (6, 43). In this study, PHF was positively associated with glucose metabolic dysfunction markers in the IFG/IGT group, as well as strongly positively correlated with the inflammatory marker CRP. Both IFV and IFV/TPV were positively associated with the inflammation markers IL-6 and CRP in the NGT group (Figure 5).

Being overweight or obese is usually strongly associated with fatty liver disease. Liver dysfunction, especially insulin resistance, is one of the key characteristics of T2DM (44, 45). Although both fatty liver and pancreas are associated with the incidence of T2DM, a study showed that fatty pancreas was an independent risk factor for diabetes in a 10-year prospective cohort study (46). Another study showed that the hazard ratio for T2DM was 3.38 for pancreatic fat in 146 patients with T2DM over 6 years of follow-up. It also showed that pancreatic fat was negatively associated with insulin secretion in participants with prediabetes but not with a metabolomic pattern (47). And another study recruited 132 T2DM patients and investigated the potential association between pancreatic fat with T2DM using CT which found that pancreatic fat was a potential predictor of beta cell dysfunction as well as T2DM (48). However, these previous studies did not distinguish the potential heterogeneous roles of different parts of pancreatic fat in glucose metabolic dysfunction. After adjusting for the liver dysfunction markers ALT, AST, and GGT in Model 2, PHF, PBF, and PTF were still positively associated with glucose metabolic dysfunction, especially in the NGT and IFG/IGT groups; whereas IFV was significantly associated with glucose metabolic dysfunction mainly in the IFG/IGT group, but not in the NGT group (Figure 5).

Inflammation played roles in the development of obesity related T2DM. Previous cross-sectional and prospective studies have indicated that CRP, IL6, IL8, IL1β, and TNFα are predictive biomarkers for T2DM (49, 50). During obese, the enlarge adipose tissue and liver could release pro-inflammation cytokines such as IL6, TNFα, and CRP to blood (49). Excessive glucose and lipids could stress pancreatic β cells and insulin responsive tissues including liver, adipose tissue and muscle which lead to overproduce cytokines. The NF-κB and JNK pathway was involved in inflammation induced insulin resistance and T2DM development (51, 52). Here, we found that Both IFV and IFV/TPV were positively associated with the inflammation markers IL-6 and CRP in the NGT group, and CRP was also positively associated with PHF in both IFG/IGT and T2DM group (Figure 5).

This study had certain limitation due to the limited ethnic origin of the participants. All the participants were from China, which may limit the generalizability of our findings. Future studies should involve patients with diverse ethnic backgrounds to validate if our findings are generalizable.

In conclusion, this study dissected the potential role of different types of pancreatic fat in overweight or obese patients with distinct diabetic status using mDixon MRI. Pancreatic fat, including PHF, PBF, and PTF, was shown to be a potential independent risk factor for the progression of diabetes, whereas IFV exacerbated glucose metabolic dysfunction. Pancreatic intra-lobular fat, including PHF, PBF, and PTF, may be a better index for the diagnosis of glucose metabolic dysfunction than inter-lobular fat. Hence, the detection of fat in different parts of the pancreas, including PHF, PBF, PTF, and IFV, may facilitate the early diagnosis of metabolic dysfunction, allow early intervention, and enable the evaluation of certain treatments on glucose metabolic dysfunction. Future studies should evaluate the roles of different types of pancreatic fat in other populations.

## Data availability statement

The original contributions presented in the study are included in the article/supplementary material, further inquiries can be directed to the corresponding authors.

## Ethics statement

The studies involving humans were approved by Medical Ethics Committee of Shanghai East Hospital. The studies were conducted in accordance with the local legislation and institutional requirements. The participants provided their written informed consent to participate in this study.

## Author contributions

LW: Investigation, Writing – original draft, Data curation, Formal analysis, Writing – review & editing. YL: Investigation, Data curation, Writing – original draft. RL: Investigation, Data curation, Software, Validation, Writing – original draft. JL: Formal analysis, Visualization, Writing – review & editing. KC: Methodology, Validation, Writing – review & editing. TL: Investigation, Writing – review & editing. HH: Investigation, Writing – review & editing. SC: Validation, Writing – review & editing. LB: Methodology, Writing – review & editing. LL: Funding acquisition, Investigation, Writing – review & editing. HW: Conceptualization, Investigation, Supervision, Writing – review & editing. QL: Conceptualization, Funding acquisition, Project administration, Supervision, Writing – review & editing.
